# A Simple, Cost-Effective Sensor for Detecting Lead Ions in Water Using Under-Potential Deposited Bismuth Sub-Layer with Differential Pulse Voltammetry (DPV)

**DOI:** 10.3390/s17050950

**Published:** 2017-04-25

**Authors:** Yifan Dai, Chung Chiun Liu

**Affiliations:** Department of Chemical & Biomolecular Engineering and Electronics Design Center, Case Western Reserve University, 10900 Euclid Avenue, Cleveland, OH 44106, USA; yxd176@case.edu

**Keywords:** under-potential deposition, bismuth sub-layer, lead ions, DPV

## Abstract

This research has developed a simple to use, cost effective sensor system for the detection of lead ions in tap water. An under-potential deposited bismuth sub-layer on a thin gold film based electrochemical sensor was designed, manufactured, and evaluated. Differential pulse voltammetry (DPV) measurement technique was employed in this detection. Tap water from the Cleveland, OH, USA regional water district was the test medium. Concentrations of lead ion in the range of 8 × 10^−7^ M to 5 × 10^−4^ M were evaluated, showing a good sensitivity over this concentration range. The calibration curve for the DPV measurements of lead ions in tap water showed excellent reproducibility with R^2^ value of 0.970. This DPV detection system required 3–6 min to complete the detection measurement. A longer measurement time of 6 min was used for the lower lead ion concentration. The selectivity of this lead ion sensor was very good, and Fe III, Cu II, Ni II, and Mg II at a concentration level of 5 × 10^−4^ M did not interfere with the lead ion measurement.

## 1. Introduction

Lead is a highly poisonous metal, both to humans and the environment. Lead poisoning of children is a major environmental health problem. The neurotoxic effect of lead and lead ions is profound, damaging the central and peripheral nervous systems resulting in stunted growth, behavioral problems, and learning disabilities. A 10 µg/dL of lead ions in blood will affect the child’s learning and behavior. A high lead level (≥70 µg/dL) can cause catastrophic health problems, including coma, seizures, and even death [1]. Lead exposure to children ages 1–5 can affect nearly every organ system in the body with increasing risks in the damage of the brain and the nervous system, resulting in slow growth, learning behavior problems, as well as hearing and speaking deficiencies [1,2]. Approximately half a million U.S. children under five years old have elevated lead levels (5 µg/dL) in blood [2,3,4]. The main exposure to lead or lead ions in humans comes from water sources. Therefore, effective protection of lead contamination is necessary, and the lead level should be able to be identified quantitatively and efficiently in both environmental and biological samples, including water sources [2,3,4].

The pollution of water sources by lead is devastating to human health. Currently, lead exists in metal water taps and interior water pipes. In addition, the corrosion of older water fixtures and solders results in lead leaching into the drinking water. Lead or lead ions are visible and can also be smelled or tasted by humans. However, the reliable method to assess lead or lead ion levels in water sources is to test the samples of the drinking water, tap water, or water sources. This assessment is time-consuming, and it requires expensive analytical instruments and skilled operators. It is typically completed by water department professionals [2,3,4]. Therefore, a simpler and more efficient detection and measurement technology for lead ions in water will be of practical and scientific importance.

Currently, analytical methods for lead determination in water sources include flame atomic absorption spectrometry (FAAS), electro-thermal atomic adsorption spectrometry (ET-AAS), inductively coupled plasma mass spectrometry (ICP-MS), inductively coupled plasma optical emission spectrometry (ICP-OES), and others. These techniques can provide accurate information about the levels of lead ion in the water sample. However, the analytical methods are elaborate and require expensive instrumentation [5,6,7,8]. Thick film screen printed carbon based modified gold sensor had been used for lead ion detection in water [9]. However, thick film screen printing employed a binder in the preparation of the printed ink. Consequently, the inclusion of the binder may have an inhibited percentage of variation in the produced sensors.

In this study, a simple-to-use, cost-effective sensor system for the detection of lead ions in water was developed. This lead ion detection sensor used a thin gold film based electrochemical sensor with a thin layer of bismuth. The bismuth layer was deposited on the gold film based electrode elements by under-potential deposition technique. Under-potential deposition provided a monolayer or sub-layer of the bismuth on the gold electrode elements enhancing the sensitivity of detecting lead ions by the bismuth film. Subsequently, the overall sensitivity of the sensor increased. Differential pulse voltammetry (DPV) was the transduction mechanism used for this sensor system. Differential pulse voltammetry (DPV) applied a linear sweep voltammetry with a series of regular voltage pulses superimposed on the linear potential sweep [10,11]. Consequently, the current is measured immediately before each potential change. Thus, the effect of the charging current is minimized, achieving a higher sensitivity. Tap water from the Cleveland, Ohio, USA regional water district was used as the test medium. Lead ion concentrations of 5 × 10^−4^ M to 8 × 10^−7^ M in tap water were tested. Potential interference studies by other metal ions including iron III, copper II, nickel II, and magnesium II at the concentration level of 5 × 10^−4^ M were carried out, demonstrating that our developed sensor processed excellent selectivity without interference by any of these metallic ions. Characterization of the bismuth was performed using Time-of-Flight Secondary Ion Mass Spectrometry (TOF-SIMS) and X-ray Photoelectron Spectroscopy (XPS). Our study showed that the total detection time for lead ions in the water was within 3 min at a lead ion concentration level of 10^−4^ M or above, and by spending 6 min for lower lead ions concentration at 10^−7^ M. This operation time could be further optimized. Furthermore, the cost of fabricating and developing this single-use sensor for lead detection was estimated to be less than $2 US dollars. Thus, a simple-to-use, cost effective practical sensor for lead ion detection in water became a true reality.

## 2. Materials and Methods

### 2.1. Apparatus and Reagents

Bismuth(III) nitrate pentahydrate (Cat. # 383074) and lead(II) nitrate (Cat. # 228621) were obtained from Sigma-Aldrich (St. Louis, MO, USA). Iron(III) sulfate pentahydrate (Cat. #AC345231000), nickel(II) sulfate hexahydrate (Cat. # N73-100), copper(II) sulfate pentahydrate (Cat. # BP346), magnesium(II) sulfate heptahydrate (Cat. # M63), sodium chloride (Cat. # S271), potassium hydroxide pellets (Cat. #P1767), concentrated H_2_SO_4_ 95.0 to 98.0 *w*/*w* % (Cat. # A300) and concentrated HNO_3_ 70% *w*/*w* % (Cat. # A200) were received from Fisher Scientific (Pittsburgh, PA, USA). A CHI 660C (CH Instrument, Inc., Austin, TX, USA) Electrochemical Workstation was used for DPV and electrochemical impedance spectroscopy (EIS) investigations. Similar Model CHI 660 A-E Electrochemical Workstations could also be used. All the experiments were conducted at room temperature. Characterization of the bismuth film was performed using a PHI TRIFT V nanoTOF Time-of-Flight Secondary Ion Mass Spectrometer (TOF-SIMS) and a PHI Versaprobe 5000 Scanning X-ray Photoelectron Spectrometer (XPS).

### 2.2. Design and Fabrication of the Sensor

This basic sensor involved a platform designed in our laboratory. It consisted of a three-electrode electrochemical configuration sensor system. Both working and counter electrodes were thin gold film of 50 nm in thickness. The thin gold film was deposited by sputtering technique on a roll-to-roll manufacturing basis. The reference electrode was a thick-film printed Ag/AgCl electrode. Laser ablation technique was used to define the size and structure of the electrode elements and the overall sensor structure. This manufacturing process employed known micro-fabrication procedures, such as sputtering physical vapor deposition, laser ablation, and thick film printing techniques, resulting in a high-reproducible and low-cost single-use disposable sensor which was very beneficial for simple-to-use, disposable, cost effective in situ applications. The overall dimensions of an individual lead ion detection sensor were 33.0 × 8.0 mm^2^. The working electrode area was 1.54 mm^2^ accommodating 10–25 µL of liquid test sample. Details of the design and fabrication of this platform thin gold-film based sensor were given elsewhere [12,13].

### 2.3. Modification and Preparation of the Lead Ions Sensor

#### 2.3.1. Chemical Pretreatment of the Thin Gold Film Electrode Elements

A three-step chemical pretreatment procedure was applied to the sensor in order to eliminate the oxidized compounds and any other residue from the gold film electrode element surface. The purpose of pretreatment was to minimize the electrode charge transfer resistance, thereby improving the sensitivity and the reproducibility of the sensor. This pretreatment procedure was based on other reported investigation [14,15] as well as in our own previous studies [12,13]. Typically, a batch of eight thin gold film based sensors were immersed in a 2 M KOH solution for 15min. After rinsing with copious amounts of DI water for about 30 s, the sensors were placed in a 0.05 M H_2_SO_4_ solution (95.0 to 98.0 *w*/*w* %) for another 15 min. DI water was then used to rinse the sensor prototypes for another 30 s. The sensors were then placed in a 0.05 M HNO_3_ solution (70% *w*/*w* %) for another 15 min. The sensors were rinsed one more time with DI water for 30 s and dried gently in a steam of nitrogen. The EIS study of this pretreated sensor showed excellent reproducibility as reported in our other studies [12,13].

#### 2.3.2. Under-Potential Deposition of Bismuth on the Thin Gold Film Based Sensor

Bismuth was considered unique for lead ion detection. Thus, bismuth-modified electrochemical based electrodes were proposed for lead ion detection in different test media [6,16,17,18,19,20,21]. One advantage of using bismuth film for the detection of lead ions was that the dissolved oxygen in the test medium did not interfere with the measurement of lead ion [22,23,24]. Thus, the use of bismuth to detect lead ions would not require removing the dissolved oxygen in the test medium which was very attractive for practical applications. Thus, bismuth film was adopted to be used in this lead ion detection approach. However, the limited sensitivity and specific required test medium condition (alkaline solution) of using a bismuth film for lead ion detection limits its practical applications in the lead ion detection in a water medium.

**Figure 1:**
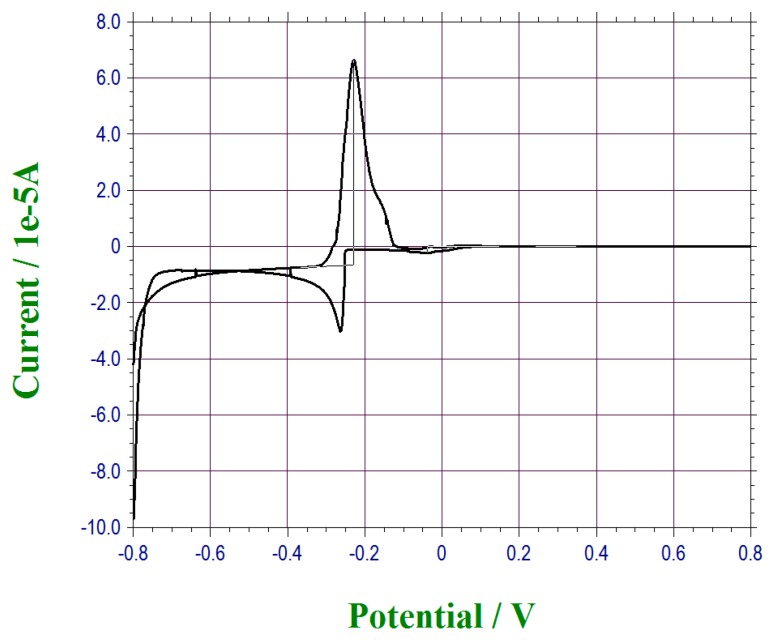
Cyclic voltammetry for the full range of scanning for bismuth reaction potential.

Under-potential deposition was a known electrochemical process. In this deposition process, the sub-layer of the selected metallic deposition was known to be extremely sensitive to the surface structure of the electrode, enhancing the sensitivity of electrode per se [25,26,27]. Our previous investigation of under-potential deposited metallic films in sub-layer thickness demonstrated the enhancement of the sensitivity of the sensor itself also, such as cadmium and thallium sub-layers for the detection of carbon dioxide and glucose, respectively [28,29]. Thus, a unique aspect of this study was that an under-potential deposited bismuth film on the thin gold film based sensor was first prepared for the lead ion detection sensor, providing the sensitivity for this lead ion detection. The under-potential deposition of bismuth might be monolayer or sub-layer. Assessment of the actual monolayer or sub-layer of bismuth film is beyond the scope of this research and will not be discussed in this study. For practical application, the under-potential deposited bismuth sub-layer provided the sensitivity of the lead ion detection sensor was our motivation in this study. In a typical preparation of this step, cyclic voltammetry was first applied for the bismuth film deposition. A bismuth ion solution was prepared. 0.025 M of Bismuth(III) nitrate pentahydrate was mixed in 1 M of nitric acid with 1 mM of sodium chloride solution. A full range of CV scan for bismuth deposition was then conducted, assessing the reduction potential for bismuth onto the gold electrode. A similar CV deposition of bismuth on gold was also conducted by Hara et al. [27]. Cathodic potentials were observed with different thicknesses of bismuth layer on the surface. For our CV study, the reduction potential of bismuth was set at −0.25 V versus the thick-film printed Ag/AgCl reference electrode as shown in Figure 1. In a typical experience, 20 µL of prepared bismuth solution was placed on the sensor and a potential sweep from −0.50 V to −0.40 V of the cyclic voltammetry was used for the under-potential deposition of the bismuth on the gold electrode. After deposition, the bismuth sensor was rinsed with deionized water for 10 s and dried gently by nitrogen. This preparation step of this under-potential deposition of bismuth film could be accomplished prior to the actual water sample testing. Figure 1 shows the typical CV used for this under-potential deposited bismuth film. Bismuth layer was visible and prepared sensors could then be stored at 4 °C, ready for use.

#### 2.3.3. Surface Characterization of Bismuth Sublayer with XPS and Tof-SIMS

The bismuth sub-layer formed by the under-potential deposition described above was examined using X-ray Photoelectron Spectroscopy and Time-of-Flight Secondary Ion Mass Spectrometry (TOF-SIMS). In this characterization study, two different bismuth concentrations were used, sample A (0.1 M) and sample B (0.25 M) of bismuth nitric solutions.

**Figure 2:**
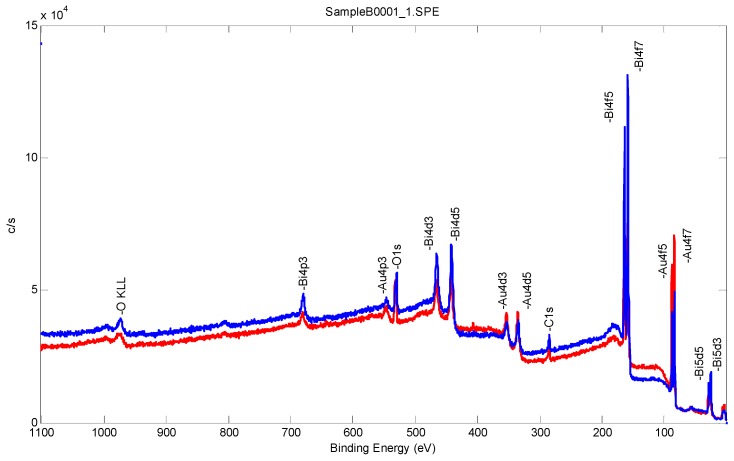
Survey scan from XPS measurements at 90 degrees take of angle comparing samples A (red) and B (blue).

**Figure 3:**
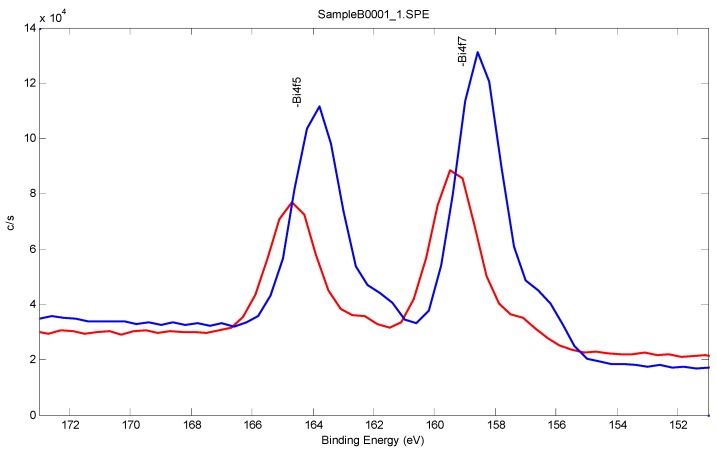
Bismuth 4d 7/2 peak from the Survey spectrum compared for samples A (red) and B (blue).

Figure 2 and Figure 3 and Table 1 show the XPS analysis of the bismuth film deposited, quantifying the bismuth film composition acquired with a monochromated aluminum K alpha X-ray with an energy of 1486.7 eV and a spot size of 200 µm. The spectrum was acquired with a 90 degree take off angle to increase the surface sensitivity. The survey scan was acquired with a Band pass of 93.9 eV. An average of eight rounds of acquisition on a range of 0 to 1100 eV was scanned every 0.4 eV for 25 ms.

Figure 2 and Figure 3 show the bismuth sublayer was successfully deposited by the under-potential deposition technique. As expected and demonstrated in Figure 3, the 0.25 M of bismuth nitric solution provided a better coverage of the gold film based sensor. Therefore, 0.25 M of bismuth nitric solution was used throughout this investigation. Table 1 shows the XPS results of the atomic percentage of the samples A and B with the higher bismuth atomic percentage in sample B.

ToF-SIMS measurements were performed in the negative polarity. At this negative polarity, bismuth and gold provided good information with a primary source of Ga. Experimentally, the primary source was a Ga^+^ beam accelerated to 30 KV and bunched to a pulse size of 7 ns and an acquisition rate of 22 KHz. Using this setting, the surface of the electrode could be mapped with a spatial resolution of 500 nm. Map stitching was then used to generate ion maps with a total area of 2 × 2 mm.

**Figure 4:**
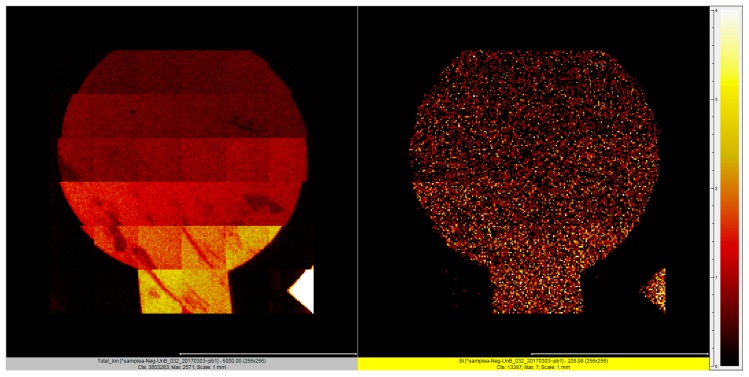
The total secondary ions acquired at the negative polarity of gold (**left**) and the bismuth ion image (**right**) using a Ga^+^ primary source.

Figure 4 shows the TOF-SIMS analysis of the under-potential deposited bismuth structure on the gold film based sensor. Figure 4a shows the gold film based working electrode element of sensor, and Figure 4b shows the bismuth film deposited on the gold based working electrode element using a 0.25 M bismuth nitric solution and at an electrochemical potential range of −0.45 V to −0.35 V vs. an Ag/AgCl thick film printed reference electrode.

The homogeneity of the bismuth film was evident. The compositions of the bismuth film deposited was mainly bismuth, as expected. Also as expected, the bismuth film deposited with the concentration of 0.25 M bismuth nitric solution was higher than that with 0.1 M bismuth nitric solution (data did not included).

## 3. Results and Discussion

### 3.1. Anodic Stripping of Lead Ions by Differential Pulse Voltammetry (DPV)

**Figure 5:**
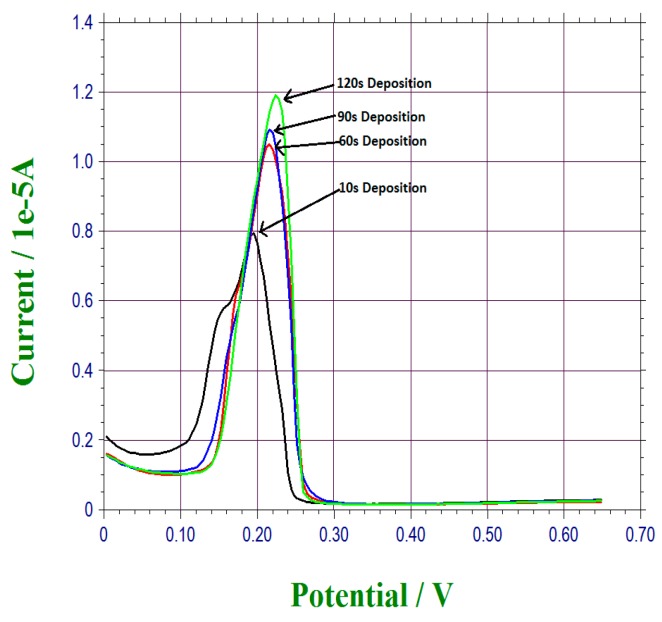
Electrochemical stripping analysis of lead ions for different deposition time ranging from 10 s to 120 s.

Lead ions deposited on the bismuth sub-layer was a time-dependent. The lead ions in the test medium reduced forming Pb(Bi) complex on the bismuth sub-layer [23,24,30,31]. The DPV measurement of the anodic stripping current of the lead ions was then used to quantify the lead ions in the test medium. As expected, the longer the time that allowed the lead ions to be attached to the bismuth sub-layer, the higher the resulting anodic stripping current measured by DPV. In this phase of the study, tap water with 8 × 10^−4^ M lead ions was used as the test medium. The period of time (the retention time) for the lead ions deposited on the bismuth sub-layer varied between 10 to 120 s. Typically, 20 µL of the test water sample was placed on top of the sensor surface. After the retention time, DPV measurement was then carried out. As expected, the highest anodic stripping current was obtained at 120 s, and the anodic stripping current was decreased in sequential order of 90, 60, and 10 s. Thus, 120 retention time was used in our detection of lead ions in tap water at this lead ion concentration level. The DPV measurement was completed in 60 s. Therefore, the overall time required for the detection of this lead ion level was within 3 min. This operating time has not been optimized and could be further modified. Figure 5 shows the results of this study.

Consequently, a fixed retention, 120 s in this case, would be used for the practical application of the lead ion detection sensor system. 

### 3.2. Detection of Lead Ions in Tap Water

**Figure 6:**
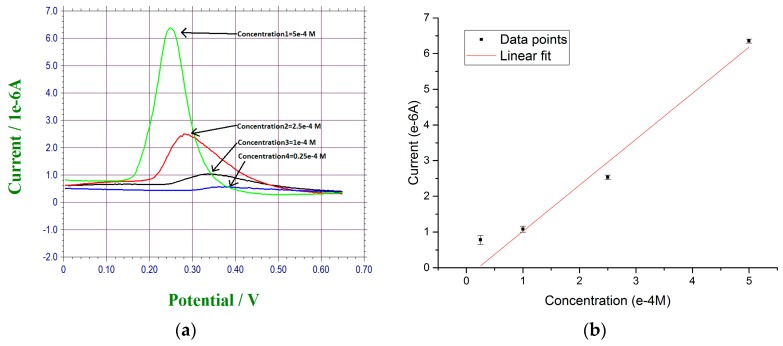
(**a**) DPV measurement of lead ions concentration level ranging from 5 × 10^−4^ M to 2.5 × 10^−5^ M; (**b**) Calibration curve for the DPV measurement data points.

Normal tap water from the Cleveland regional water district, Cleveland, OH. USA, was used as the test medium for this lead ion sensor detection. Lead(II) nitrate in proper quantity was added into the tap water preparing for the lead ions contained tap water test sample. In a typical experiment, 20 µL of the test water sample was placed on top of an under-potential deposited bismuth sub-layer thin gold film based sensor. At this 10^−4^ M lead ions concentration, a retention time period of 120 s was used based on the results described in Section 3.2. After this retention time, DPV measurement of the anodic stripping current was then undertaken. Figure 6a shows the DPV measurement of four lead ions concentrations in the tap water, namely, 5 × 10^−4^ M, 2.5 × 10^−4^ M, 1 × 10^−4^ M, and 0.25 × 10^−4^ M with n > 3. Potential shifts were observed in our DPV measurements. The stripping reaction of lead from the bismuth layer is an irreversible reaction. Faradic current, a diffusional control reaction influenced by concentration difference, was then measured by DPV technique. Also, experimental parameters, including the electrode reaction rate constant, transfer coefficient, waveform parameters, affected DPV measurement [32,33]. Consequently, the minor potential shift of the DPV waveform was due to these factors. Figure 6b shows the calibration curve based on the DPV measurements obtained in Figure 6a. The least square fit of this calibration curve is Y = 1.16X − 0.02 with R^2^ value of 0.970. The results of this test demonstrated that the accuracy of detecting lead ions in tap water using this under-potential deposited bismuth sublayer thin gold film based electrochemical sensor with DPV measurement was very good. The retention time was 120 s and the DPV measurement time was 60 s. Thus, the total time for the detection of lead ions could be accomplished within 180 s (3 min).

**Figure 7:**
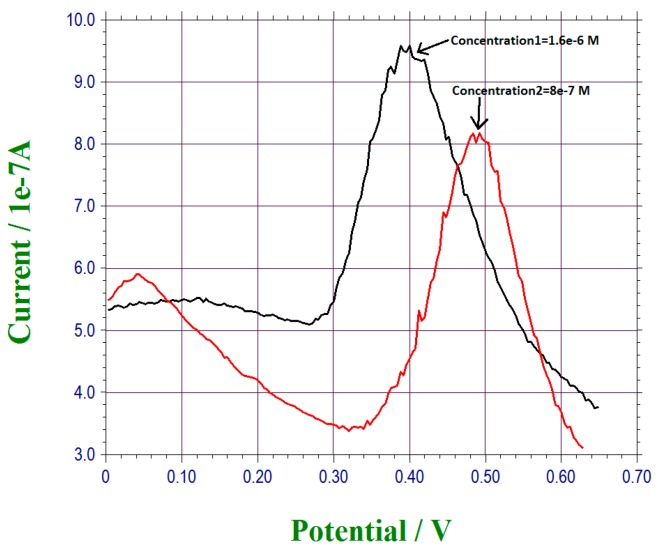
DPV measurement of lead concentration levels of 1.6 × 10^−6^ M and 8 × 10^−7^ M.

Lower lead ion concentrations could be detected using this under-potential deposited bismuth sub-layer on this thin gold film based sensor and with DPV measurement. As suggested in Section 3.2, the rate at which lead ions adhered to the bismuth sub-layer was a time-dependent function. Experimentally, a tap water sample containing a low level of lead ions (20 µL) was placed on top of the sensor identical to the process described above. However, at this lower lead ions concentration level, the retention time was set at 300 s (5 min), and DPV measurement was then carried out. It also required 60 s to complete the DPV measurement. Figure 7 shows the DPV measurement of lead ions in tap water with a lead ion concentrations of 1.6 × 10^−6^ M and 8 × 10^−7^ M. It was apparent that this under-potential deposited bismuth sub-layer on a thin gold film sensor was capable of detecting lead ions in tap water as low as 8 × 10^−7^ M. The detection process required a total of 360 s (6 min) using the DPV measurement technique.

Based on the results shown in Figure 6 and Figure 7, a retention time of 300 s would cover the lead ions concentration range of 8 × 10^−7^ M to 5 × 10^−4^ M. The retention time needed for the lead ion detection can be further optimized.

### 3.3. Interference Study of This Lead Ions Sensor

**Figure 8:**
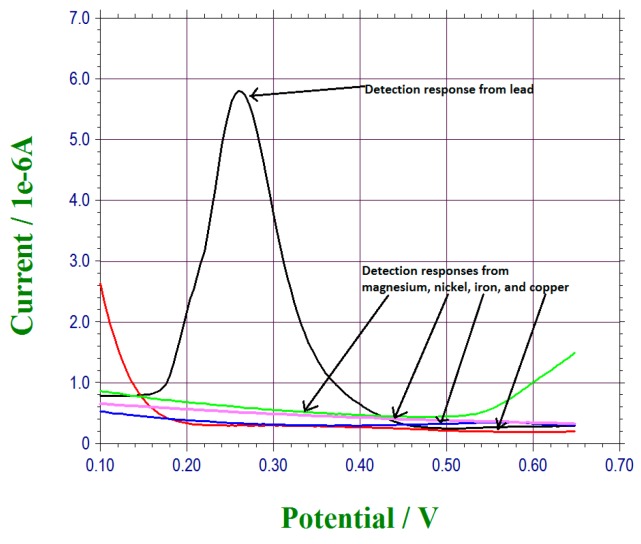
Interference test for the bismuth sensor.

Selectivity is very important for the development of a sensor. This suggested that the operation of the sensor should not interfere with other chemicals that might be presented in the test medium. Fe III, Cu II, Ni II, and Mg II and were considered to be carcinogens and may also interfere with lead bismuth reaction [34,35,36,37]. Iron sulfate pentahydrate, cooper sulfate pentahydrate, nickel sulfate hexahydrate, and magnesium sulfate heptahydrate were used. Each of these chemicals was used to prepare a 5 × 10^−4^ M concentration test sample with the Cleveland regional water district tap water. Similar to the test procedure described above, 20 µL of the water sample was placed on top of a prepared lead ion sensor. The retention time was set at 300 s ensuring that the metal ions were anodic stripped from the bismuth sub-layer. DPV measurement was undertaken after the retention time 300 s. Similar to our standard test, the DPV measurement took 60 s. Figure 8 shows that none of the Fe III, Cu II, Ni II, and Mg II at 5 × 10^−4^ M in tap water contribute to the DPV measured current of 5 × 10^−4^ M lead ions in the tap water sample. This result indicated the excellent selectivity of the under-potential deposited bismuth sub-layers of a thin gold film based sensor for lead ion detection.

## 4. Conclusions

A unique lead ion detection sensor system was successful developed. This sensor used an under-potential deposited bismuth sub-layer on a thin gold based electrochemical sensor. Differential pulse voltammetry (DPV) was the transduction mechanism employed in this detection system. Lead ions in normal tap water from the Cleveland regional water district were used as the test medium. Lead ion concentrations of 5 × 10^−4^ M to 8 × 10^−7^ M were detected within 3–6 min including the retention and DPV measuring times. Fe III, Cu II, Ni II, and Mg II at 5 × 10^−4^ M concentration in tap water samples did not contribute to the DPV current output of lead ions detection, indicating the sensor was highly selective to lead ions without other interference.

## Figures and Tables

**Table 1:** Concentration of different elements (in Atomic Percent) compared for samples A and B.

Sample No.	C1s	O1s	Au4f	Bi4f
Sample A	31.1	40.7	15.2	13.0
Sample B	27.5	40.5	10.3	21.8
